# Isobutylamine, Isopropylamine, *sec*-Butylamine, Propylamine, Hexylamine, Pentylamine and 2-Methylbutylamine (Flavoring Substances)

**DOI:** 10.14252/foodsafetyfscj.D-1900003

**Published:** 2019-06-28

**Authors:** Food Safety Commission of Japan

## Abstract

Food Safety Commission of Japan (FSCJ) conducted risk assessments of isobutylamine, isopropylamine, *sec-*butylamine, propylamine, hexylamine, pentylamine and 2-methylbutylamine, which are used as food additives (flavors) (hereinafter, referred to as “the flavoring agents”), based on the Guidelines for the Assessment of Flavoring Substances in Foods on Health (Decision of the Commission Dated May 2016, hereinafter, referred to as the Guidelines on Flavoring Substances), using various documents. Based on the structural and metabolic similarity, FSCJ regarded that the identical procedures are applicable for the risk assessments of all the flavoring agents. FSCJ judged that the seven flavoring agents have no genotoxicities relevant to human health on the basis of the evaluation of analogous compounds. FSCJ metabolized to innocuous products with no food safety concerns. The estimated daily intakes of all the flavoring agents are within the range of 0.02 μg/person per day to 2 μg/person per day, which are below the threshold of concern (i.e., 1,800μg/person per day for Class I), and therefore, FSCJ judged that the flavoring agents are considered to be of no concern for food safety. In summary, FSCJ concluded, as a result of the safety assessment, that there is no safety concern with the flavoring agents, isobutylamine, isopropylamine, *sec*-butylamine, propylamine, hexylamine, pentylamine, and 2-methylbutylamine, as long as they are used as flavorings in foods.

## Conclusion in Brief

Food Safety Commission of Japan (FSCJ) conducted risk assessments of isobutylamine (CAS No. 78-81-9), isopropylamine (CAS No. 75-31-0), *sec*-butylamine (CAS No. 13952-84-6), propylamine (CAS No. 107-10-8), hexylamine (CAS No. 111-26-2), pentylamine (CAS No. 110-58-7) and 2-methylbutylamine (CAS No. 96-15-1), which are used as food additives (flavors) (hereinafter, referred to as “the flavoring agents”), based on the Guidelines for the Assessment of Flavoring Substances in Foods on Health (Decision of the Commission Dated May 2016, hereinafter referred to as the Guidelines on Flavoring Substances), using various documents.

Based on the structural and metabolic similarity, FSCJ regarded that the identical procedures are applicable for the risk assessments of all the flavoring agents.

FSCJ judged that the seven flavoring agents have no genotoxicities relevant to human health on the basis of the evaluation of analogous compounds.

FSCJ judged that the flavoring agents, belonging to structural Class I, are metabolized to innocuous products with no food safety concerns. The estimated daily intakes of all the flavoring agents are within the range of 0.02 μg/person per day to 2 μg/person per day, which are below the threshold of concern (i.e., 1,800μg/person per day for Class I), and therefore, FSCJ judged that the flavoring agents are considered to be of no concern for food safety.

In summary, FSCJ concluded, as a result of the safety assessment, that there is no safety concern with the flavoring agents, isobutylamine, isopropylamine, *sec-*butylamine, propylamine, hexylamine, pentylamine, and 2-methylbutylamine, as long as they are used as flavorings in foods.

